# Editorial for the Special Issue on 3D Printing for Tissue Engineering and Regenerative Medicine

**DOI:** 10.3390/mi11040366

**Published:** 2020-03-31

**Authors:** Vahid Serpooshan, Murat Guvendiren

**Affiliations:** 1 Department of Biomedical Engineering, Emory University School of Medicine and Georgia Institute of Technology, Atlanta, GA 30322, USA; 2 Department of Pediatrics, Emory University School of Medicine, Atlanta, GA 30322, USA; 3 Children’s Healthcare of Atlanta, Atlanta, GA 30322, USA; 4 Otto H. York Chemical and Materials Engineering, New Jersey Institute of Technology, Newark, NJ 07102, USA; 5 Department of Biomedical Engineering, New Jersey Institute of Technology, Newark, NJ 07102, USA

Three-dimensional (3D) bioprinting uses additive manufacturing techniques to fabricate 3D structures consisting of heterogenous selections of living cells, biomaterials, and active biomolecules [1,2]. To date, 3D bioprinting technologies have transformed the fields of tissue engineering and regenerative medicine by enabling fabrication of highly complex biological constructs. Using the patient’s medical imaging data, patient- and damage- specific implants can be printed with customized cellular and physiomechanical functionalities [3,4,5]. The main bioprinting methods include extrusion-based, droplet-based (inkjet), laser-based, and, more recently, vat photopolymerization-based bioprinting [6,7]. A variety of biomaterials (i.e., *bioinks*) have been used for tissue bioprinting, including ceramics, synthetic and natural polymers, decellularized tissues, and more frequently, hybrid bioinks consisting of a combination of these materials [8,9,10,11].

While significant and rapid progresses have been made in tissue bioprinting processes for various in vitro applications, such as disease modeling [12] and drug screening [13], there are several challenges to address before bioprinting becomes clinically relevant [14,15,16]. These constraints include: 1) limited number of available bioink solutions and lack of thorough characterization of their biological and physiomechanical properties [10,17]; 2) poor understanding of the correlation between printed architecture and the ultimate tissue function [18,19]; 3) limitations on the quality of imaging techniques [20,21] and available bioprinters [22]; 4) complex and rather expensive processes involved pre, during, and post-bioprinting [22]; 5) suboptimal, non-specialized printing software and their often incompatibilities [23].

There are eight articles published in this Special Issue composed of four research papers and four review papers. The research articles focus on the influence of electron beam (E-beam) sterilization on in vivo degradation of composite filaments [24], enhancing osteogenic differentiation of stem cells using 3D printed wavy scaffolds [25], the development of a scaffold-free bioprinter [26], and the fabrication of multilayered vascular constructs with a curved structure and multi-branches [27]. Kang et al. investigated the effect of E-beam sterilization on the degradation of β-tricalcium phosphate/polycaprolactone (β-TCP/PCL) composite filaments in a rat subcutaneous model for 24 weeks [24]. Although they reported that the E-beam sterilization accelerated the degradation rate of the composite filaments, due to the decreased crystallinity and decreased molecular weight of PCL after the E-beam irradiation, they concluded that the chemistry of samples plays a bigger role than the sterilization method in biodegradation. Ji and Guvendiren investigated the effect of wavy scaffold architecture on human mesenchymal stem cell (hMSC) osteogenesis by 3D printing as compared to orthogonal scaffold design [25]. They found that when cultured on wavy scaffolds, hMSCs became elongated, formed mature focal adhesions, and showed significantly enhanced osteogenesis. LaBarge et al. developed a custom device enabling the printing of an entire layer of spheroids at once to reduce printing time [26]. They demonstrated the feasibility of this device first using zirconia and alginate beads, which mimic spheroids, and human-induced pluripotent stem cell-derived spheroids. This scaffold-free bioprinter could potentially advance the growing field of scaffold-free 3D bioprinting. Liu et al. developed a combined approached to fabricate multilayered biodegradable vascular constructs for cardiovascular research [27]. In their approach, 3D printing was used to fabricate a mold system which was then used to cast a hydrogel and a sacrificial material. They investigated the channel wall displacement during blood flow using fluid-structure interaction simulations. They also demonstrated the feasibility of their devices using human umbilical vein endothelial cells. Their approach shows a great potential for constructing integrated vasculature for tissue engineering.

The four review articles focused on advanced polymers for 3D organ printing [28], chitosan for tissue and organ bioprinting [29], applications of 3D printing for craniofacial tissue engineering [30], and in vivo tracking of 3D printed tissue-engineered constructs [31]. Wang reviewed advanced polymers exhibiting excellent biocompatibility, biodegradability, 3D printability and structural stability [28]. The author also summarized the challenges of polymers for 3D bioprinting of complex organs. Li et al. reviewed the use of chitosan in tissue repair, including skin, bone, cartilage, and liver tissue, and 3D bioprinting of organs [29]. Tao et al. focused on the applications of 3D printing for craniofacial tissue engineering, including periodontal complex, dental pulp, alveolar bone, and cartilage [30]. Gil et al. reviewed the currently utilized imaging techniques to track tissue engineering scaffolds in vivo, with particular focus on the in vivo tracking of 3D bioprinted tissue constructs [31].

We would like to take this opportunity to express our gratitude to all authors who contributed to this Special Issue. We also wish to thank all the reviewers for dedicating their time to provide thorough and timely reviews to ensure the quality of this Special Issue.
